# Editorial to the Special Issue “Acoustic Sensing and Monitoring in Urban and Natural Environments”

**DOI:** 10.3390/s24196295

**Published:** 2024-09-29

**Authors:** Hector Eduardo Roman

**Affiliations:** Department of Physics, University of Milano-Bicocca, Piazza della Scienza 3, 20126 Milano, Italy; hector.roman@unimib.it

During the last decades, the great advances achieved in sensor technology and monitoring strategies have been instrumental to accurately quantify anthropogenic noise pollution in both urban and natural environments. Indeed, when lacking of human influence, a natural habitat soundscape constitutes a benchmark which allows us to estimate any negative impact that anthropogenic activity can have in a particular surrounding. The study of natural soundscapes can therefore be very useful for the development of new ideas and methodologies aimed at improving the quality of life in highly populated urban areas which may suffer from self-produced noise pollution. The latter is one of the greatest environmental threats to people’s health, which has become an issue affecting millions of people worldwide.

Historically, human society develops more efficiently in urban areas which facilitate its great variety of activities. The emergence of large populated zones, however, is not the result of a long-term planning and therefore they are not optimized to yield high living standards. As a result, people living in large conglomerates must cohabit with the often deleterious actions of different agents, among which anthropogenic noise plays a central role. Only recently these issues have been considered seriously yielding the development of new branches of study and research. The latter are essential to guide and support efficient policy-making decisions aimed at finding the right intervention measures.

The aim of this Special Issue is to gather experts actively working in different fields of acoustic phenomena, such as sensing and monitoring techniques in either urban or natural environments, to help expanding our knowledge on soundscape monitoring and analysis contributions 1–7, road traffic and soundscape modeling contributions 8–10, and the development of more efficient sensors contributions 11–14 in support of such different endeavours.

In contribution 1, ‘Hearing to the Unseen’, Bota et al. present a work which can significantly improve on standard passive acoustic sensing (PAS) and environmental monitoring, the latter known to be challenging when huge amount of data needs to be collected. Specifically, they develop a new technique able to recognize species-specific sounds more efficiently, denoted as BirdNET, based on a novel machine learning tool for automated recognition and acoustic data processing. They apply BirdNET for detecting two cryptic forest bird species: Coal Tit (*Peripatus ater*) and the Short-toed Treecreeper (*Certhia brachydactyla*), illustrating the achievement of highly accurate recognition rates, typical of BirdNET performance. In addition, the software has been made freely available, encouraging researchers and managers to utilize it.

Understanding the impact of urbanization on the surrounding biodiversity and wildlife conservation is a main ecological issue. Often, the actual impact of a rapid urbanization is difficult to assess using traditional PAS methods, as discussed by Barnes and Quinn in contribution 2. They present a multimethod analysis of biodiversity in the rapidly urbanizing county Greenville (South Carolina, USA), based on several audio recodings in 25 locations along a predertermined trail, supplemented with visual assessments, an online database and the use of refugia tubes. The local species identification along the trail was employed to identify relationships between herpetofauna and acoustic indices (as proxies for biodiversity), suggesting that the use of different sampling methods is crucial for achieving a more comprehensive and realistic evaluation of wildlife occupancy. This study should be of help to establish better conservation policies of biodiversity and appropriate urbanization guidelines in natural forested ecosystems.

Soundscape indices have been introduced with the aim of evaluating different contributions of the environmental sound components, providing accurate assessments of the “acoustic quality” within a complex habitat. In contribution 3, Benocci et al. apply machine learning (ML) algorithms (decision tree, random forest, adaptive boosting, and support vector machine) to optimize the (four) parameters determining the behavior of the soundscape ranking index (SRI). The latter was previously introduced by the authors to study sound recordings taken at 16 sites, distributed over a regular grid covering an area of about 22 hectares, within an urban park in the city of Milan surrounded by a variety of anthropophonic sources. The recordings span 3.5 h each over a period of four consecutive days. The authors find that two ML algorithms (decision tree and adaptive boosting) yield a set of parameters displaying a rather good classification performance (F1-scores: 0.70 and 0.71, respectively). The method is expected to prove useful when considering large amount of sound data, allowing for an efficient classification of the associated indices.

The above results are in quantitative agreement with a self-consistent estimation of the mean SRI values discussed in contribution 4. In the latter, a careful aural survey from a single day was performed in order to identify the presence of 19 predefined sound categories, within one minute of recording, for a total of 70 one-minute intervals. The resulting one minute histograms of the SRI values were used to define a dissimilarity function for each pair of sites. Dissimilarity increases significantly with the inter-site distance in real space, and optimal values of the 4 parameters were obtained by minimizing the standard deviation of the data, requiring consistency with a fifth parameter describing the power-law variation of dissimilarity with distance. This study can be useful to assess the quality of a soundscape in more general situations.

Predicting traffic noise over city-wide scales is challenging due to the relatively large amount of input information required to reach a sufficient accuracy. In contribution 5, the authors present a methodology by just relying on street categorization and a city microphone network. A simplified dynamic traffic model is employed to predict statistical and dynamical noise indicators, and to estimate the number of noise events. A standard sound propagation module is then employed to determine the noise levels. Finally, an ML technique elaborates the deterministic predictions of the different traffic parameter scenarios, choosing the one yielding the best accord with the indicators measured by the microphone network. The method is illustrated using data from the city of Barcelona, yielding results within a 2–3 dB precision, and number of events within a 30% accuracy. The current methodology allows considering a wide variety of noise indicators thus improving environmental noise assessment over city-wide scales.

A significant change in urban soundscapes occurred during the COVID-19 pandemic as a result of the imposed mobility and activity restrictions in the affected zones, yielding a dramatic reduction in noise pollution levels. The latter was measured in Barcelona, both before and during the lockdown, by means of a deployed acoustic sensor network consisting of 70 sound recording units, as discussed in contribution 6. Different noise indices were compared, together with a perceptual test conducted within the project Sons al Balcó during the lockdown. The analysis was based on a clustering procedure, separating objective from subjective data according to the predominant type of noise sources present in each sensor area. The areas were then classified as heavy, moderate and low-traffic areas. A reduction in noise indices values was found to be significantly correlated with an improved acoustic satisfaction and type of noise sources. These results suggest that objective calibrated data can be useful to estimate the subjective perception of urban soundscapes in cases lacking of input information.

To efficiently deal with major health and social issues in complex noisy environments, dynamical noise maps are generally needed to keep track of noise behavior in real time. An alternative approach to existing real time recording methods has been proposed in contribution 7 based on the direct use of smartphones carried by mobile individuals as measurement tools of local noise levels. To successfully implement this methodology, an accurate calibration of the smartphones is required. The authors discuss the so called “blind calibration” procedure, in which measured noise levels from several smartphones, located within a limited area, are sent to a data center for a consistent evaluation of their values, allowing for their calibration. The article proposes to set up a blind calibration method by using data from the NoiseCapture (NC) smartphone application, and tests using NC datasets are discussed in support of the suggested model.

The accurate assessment of traffic noise levels in a given urban area is a primary requisite to complay with present road traffic noise (RTN) regulations. In contribution 8, Rossi et al. propose a new methodology based on an easy application of RTN models, without the need of relying on measured data for calibration in the first place. Equivalent continuous sound pressure levels were obtained using different emission models coupled to a sound propagation algorithm, by randomly generating traffic flows, speeds, and source–receiver distances. Finally, a multilinear regressive technique was developed to deal with the data in a managable way for applications. The procedure was validated using a set of long-term traffic and noise data, recorded using several sensors (sound level meters), car counters, and speed detectors, in Saint-Berthevin (France). The estimations provided by the multilinear regressions are in very good agreement with the field measurements, displaying mean absolute errors in the range (1.60–2.64) dB(A), suggesting that RTN models can be successfully integrated into a network of traffic sensors to forecast noise levels accurately.

Railway transit plays a dominant role in exerting vibration pollution in urban areas. A common technique employed to dissipate the associated waves consists in the infilling of periodic narrow excavation dugs in the soil located along the expected wave propagation paths. However, the presently used infilled ditches do not provide sufficient attenuation at low and medium frequencies. In contribution 9, Gao et al. suggest a novel solution to cope with the most disturbing lower frequencies. The new method is based on the use of acoustic metamaterials consisting of a periodic infilled trench combined with a wave-impeding block, typically embedded at a certain depth in the ground. The authors develop a 3D finite elements model to quantify the expected isolation performance of the suggested wave barriers. The results are very promising, suggesting that the combination of periodic infilled trench structures with a wave impedance block barrier can effectively attenuate the most disturbing low and medium frequencies, helping to mitigate environmental vibrations pollution.

Environmental sound studies gain considerable insight if an accurate classification of the different type of sound sources can be accomplished. In contribution 10, Castro-Ospina et al. consider the interesting idea of representing audio data using the language of graph theory, and apply it to the problem of sound classification. The new methodology is based on the use of pre-trained audio models to extract hidden features from audio files, information which is then represented as nodes of connected graphs. To solve multi-class audio classification problems, the graphs are trained using three graph neural networks (GNNs): graph convolutional networks, graph attention networks (GATs) and GraphSAGE. The GAT model emerges as the best performer yielding an accurate classification of environmental sounds, and an excellent identification of the type of land cover such as forest, savanna, or pasture. The study embodies a promising scenario to the use of learning techniques in the context of GNNs methodology for analyzing audio data related to environmental issues.

Autonomous recording units are widely used to record vocalizing species localized either in an indoor or outdoor context, of which AudioMoth is one of the most popular ones. Despite its extensive use, few quantitative tests of AudioMoth performance have been reported. Clearly, further information on the device is needed for the design of efficient field surveys, and for processing the recording data more accurately. In contribution 11, two types of tests on AudioMoth recorder performance characteristics are reported. In the first one, settings regarding different orientations and mounting conditions, using several devices, yielded little variations in acoustic performances. In particular, protecting plastic bags used in outdoor setups have little effect on sensitivity for frequencies below 10 kHz. It is found that AudioMoth has an almost constant on-axis response, displaying some attenuation effects from sources located behind it. The latter can be of relevance when the device is positioned on a tree. In the second test, a variety of recording frequencies, gain settings, environmental temperatures, and battery types were considered. In particular, the lifespan of different types of batteries were measured, showing that lithium batteries work very well lasting for about 190 h at room temperature and sampling rate of 32 kHz, while at freezing temperatures they keep collecting data for a period twice as long as compared to their standard alkaline counterparts. This information should be of help to researchers performing lasting measurements under very different environmental conditions.

The work in contribution 12 deals with learning transfers from a ‘teacher model’ to a smaller ‘student model’. Such distillation techniques are used to design efficient, lightweight student models for speech detection in bioacoustics. Taken the EcoVAD voice detection architecture as the teacher model, a comparative analysis is perfomed on the MobileNetV3-Small-Pi model aimed at working as a compact student architecture. Various configurations of the student models were analysed to identify the optimal performance, and different distillation techniques were studied to find the most effective method of model selection. The obtained distilled models exhibit comparable performances to the EcoVAD one, suggesting a possible approach to overcome present computational barriers for real-time ecological monitoring using compact devices.

In contributions 13 and 14, Ivancic et al. report recent developments of a small, lightweight, portable sensor well adapted to resolve quiet or distant acoustic sources and their location, in addition to underwater operations. The new acoustic device is based on a micro-electromechanical system (MEMS), and a novel design of a related acoustic vector sensor array (AVS) is discussed in contribution 13. In contribution 14, extensions of the MEMS acoustic sensor are presented to broaden the operating bandwidth by keeping a high signal-to-noise ratio, in particular at low frequencies. The new approach represents a significant improvement in sensor performance compared to standard MEMS sensor ones, in which the multi-resonant design plays a fundamental role to overcome the limitations of the standard devices which increase sensitivity at the expense of bandwidth.

